# Better continuity of care improves the quality of end-of-life care among elderly patients with end-stage renal disease

**DOI:** 10.1038/s41598-020-76707-w

**Published:** 2020-11-12

**Authors:** Annie Y. Chen, Bradley Chen, Chin-Chi Kuo

**Affiliations:** 1 RAND Corporation, Santa Monica, CA USA; 2 Pardee RAND Graduate School, Santa Monica, CA USA; 3 Institute of Public Health, Linong St., National Yang Ming University, No. 155, Sec. 2, Taipei City, Taiwan, ROC; 4 Division of Nephrology, Department of Internal Medicine, China Medical University Hospital and College of Medicine, China Medical University, Taichung, Taiwan, ROC; 5 Big Data Center, China Medical University Hospital and College of Medicine, China Medical University, 2, Yude Rd, Taichung City, Taiwan, ROC

**Keywords:** Health policy, Kidney diseases, Palliative care

## Abstract

Continuity of care (COC) has been emphasized in research on terminal cancer patients to increase the quality of end-of-life care; however, limited research has been conducted on end-stage renal disease patients. We applied a retrospective cohort design on 29,095 elderly patients with end-stage renal disease who died between 2005 and 2013. These patients were identified from the National Health Insurance Research Database of Taiwan. The provider Continuity of Care Index (COCI) and site COCI were calculated on the basis of outpatient visits during the 6–12 months before death. We discovered that increases in the provider COCI were significantly associated with reductions in health expenditures after adjusting for confounders, especially in inpatient and emergency departments, where the treatment intensity is high. Higher provider and site COC were also associated with lower utilization of acute care and invasive treatments in the last month before death. Provider COC had a greater effect on end-of-life care expenditures than site COC did, which indicated significant care coordination gaps within the same facility. Our findings support the recommendation of prioritizing the continuity of end-of-life care, especially provider continuity, for patients with end-stage renal disease.

## Introduction

With an increase in life expectancy and the burden of chronic disease, increasing emphasis has been placed on the quality of care and on the need for evidence to guide quality end-of-life care^1^. Numerous studies have reported on the quality of care and its determinants^2–4^. However, as the paramount objective for patients with a terminal illness is no longer to cure but to provide comfort and ensure that the patients maintain their dignity^5^, the conceptualization and emphasis of “quality” and its determinants differ from those of typical medical care.

Despite the increasing recognition of the importance of end-of-life care, research and efforts to improve end-of-life care have predominantly focused on cancer patients^6–8^, which is reflected in the Clinical Practice Guidelines for Quality Palliative Care, 4th edition, published by the National Coalition for Hospice and Palliative Care in September 2018^9^. These guidelines are primarily based on studies in cancer and cardiovascular disease patients. The understanding and evidence on end-of-life care for other complex chronic diseases, such as end-stage renal disease (ESRD), is relatively limited. A deeper understanding of end-of-life care is required to improve its quality.

Patients with ESRD require renal replacement therapies to sustain life, which results in a high risk of adverse effects and symptomatic burdens that are similar or higher than those of cancer patients^10,11^. However, research on end-of-life care for patients with ESRD has suggested that patients with ESRD are more likely to experience aggressive end-of-life treatments than patients with cancer are^12^ and thus receive an inferior quality of care^6^. Taiwan has the highest rates of ESRD incidence and prevalence in the world^13^, and it has been striving to manage the increasing financial and societal burden of patients with ESRD on the health care system. In 2009, the National Health Insurance Administration (NHIA) expanded the reimbursement of hospice palliative care, which previously only included cancer patients, to include patients with ESRD. However, a study demonstrated that the hospice palliative care enrollment rates of noncancer patients remained substantially lower than those of cancer patients^14^.

Continuity of care (COC), which is often defined as a relationship between providers and patients after a disease episode, has long been reported to be a central driver of quality of care^15^. Studies have determined that higher COC is associated with reductions in hospitalizations, emergency department visits^16^, and costs of care^17^. Furthermore, studies investigating the effects of COC on end-of-life care in cancer patients have reported that COC has a positive effect on reducing acute care outcomes^18,19^. Given the equally high demand for COC among patients with ESRD, we hypothesized that COC is a major factor influencing the quality of end-of-life care in the ESRD population.

The universal coverage of national health insurance and the copayment waiver for patients with ESRD has enabled good access to renal replacement therapy in Taiwan. However, the removal of financial barriers and the complete freedom in the choice of physicians could lead to doctor shopping and low care coordination across providers in certain patients. Consequently, a wide variation is observed in COC, and multiple aspects of COC can be improved in Taiwan^15,20^. Taiwan presents a unique empirical case to investigate the role COC in the quality of end-of-life services because of the high disease burden of ESRD, the demand for quality end-of-life care, and the health care system structure.

The purpose of this research was to examine and provide quantitative evidence of the effects of COC on end-of-life care among patients with ESRD. The findings from our analyses could contribute to achieving high-quality end-of-life care in Taiwan and may be informative for other countries facing similar challenges. The increased spending and intensity of care at the end of life for patients with ESRD remains a critical topic that requires further investigation.

## Results

### Cohort characteristics

The study population comprised 29,095 individuals with a mean age at death of 76.58 years. The main physicians, defined as the physician a patient visited the most in the 6–12 months before death, were predominantly nephrologists (67.65%). Regional hospitals were the most common main hospital (hospital visited the most in the 6–12 months before death) among patients with ESRD (Table 1).

**Table 1:** Descriptive statistics of the study population.

**Individual characteristics**		
Age at death, NO. (%)	≥ 65, < 75	12,850 (44.17%)
	≥ 75, < 85	12,700 (43.65%)
	≥ 85	3545 (12.18%)
Age at death, mean (SD)		76.58 (6.78)
Sex, NO. (%)	Female	15,552 (53.45%)
	Male	13,543 (46.55%)
Insurable earnings, mean (SD)		TWD$22,158.66 (19,912.60)
Charlson Comorbidity Index, mean (SD)		5.52 (2.54)
Year of death, NO. (%)	2005	1977 (6.79%)
	2006	2425 (8.33%)
	2007	2798 (9.62%)
	2008	2976 (10.23%)
	2009	3162 (10.87%)
	2010	3526 (12.12%)
	2011	3843 (13.21%)
	2012	4037 (13.88%)
	2013	4351 (14.95%)
**Main physician characteristics**		
Specialty of main physician, NO. (%)	Nephrologist	19,682 (67.65%)
	Internal medicine	3941 (13.55%)
	Family medicine	922 (3.17%)
	Surgeon	1799 (6.18%)
	Others	2751 (9.46%)
Physician’s age mean (SD)		45.95 (7.97)
Physician’s sex, NO. (%)	Female	2561 (8.08%)
	Male	26,534 (91.20%)
**Main hospital characteristics**		
Primary hospital—accreditation, NO. (%)	Medical centers	5497 (18.89%)
	Regional hospitals	9165 (31.50%)
	Local hospitals	7135 (24.52%)
	Local clinics	7273 (25.00%)
	Homecare	25 (0.09%)
Primary hospital-ownership, NO. (%)	Public	5236 (18.00%)
	Private	22,859 (82.00%)
Teaching hospital, NO. (%)	Teaching	16,011 (55.03%)
	Non-teaching	13,084 (44.97%)
Region, NO. (%)	Taipei	8591 (29.53%)
	Northern	4165 (14.32%)
	central	5670 (19.49%)
	Southern	5030 (17.29%)
	Kao-Ping	4943 (16.99%)
	Eastern	696 (2.39%)
**Continuity of Care Index, 1 year before death—6 months before death**		
Continuity of Care Index—provider, mean (SD)		0.27 (0.20)
Continuity of Care Index—site, mean (SD)		0.59 (0.27)
**Outcome variables**		
**Expenditure**		
6 months before death, mean (SD), TWD$	Total	535,846.3 (345,817.3)
	Total inpatient	307,159.7 (348,130)
	Total outpatient	265,582 (85,490.37)
	Outpatient—emergency	16,576.08 (22,320.34)
	Outpatient—non-emergency	249,006 (82,787.03)
3 months before death, mean (SD), TWD$	Total	297,291.9 (244,111.7)
	Total inpatient	209,765.3 (244,152.7)
	Total outpatient expenditure	113,951.9 (53,976.16)
	Outpatient—emergency	11,231.94 (16,293.82)
	Outpatient—non-emergency	102,720 (50,821.22)
**Hospice palliative care**		
Hospice palliative care, NO. (%)		529 (1.82%)
**Acute CARE in the last month**		
ICU admission, NO. (%)		14,336 (49.27%)
ER visit, NO. (%)		15,863 (54.52%)
**Invasive treatments in the last month**		
Surgical intervention, NO. (%)		8260 (28.39%)
Ventilator, NO. (%)		13,424 (46.14%)
CPR, NO. (%)		5718 (19.65%)
Endotracheal intubation, NO. (%)		8992 (30.91%)
Continuous Renal Replacement Therapy, No. (%)		1834 (6.30%)
Nasogastric intubation, NO. (%)		18,262 (62.77%)

*SD* standard deviation, *NO* number, *ICU* intensive care unit, *ER* emergency room, *CPR* cardiopulmonary resuscitation.

**Figure 1:**
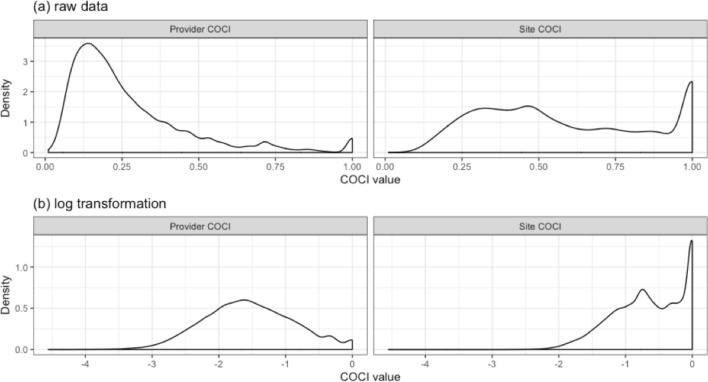
Density plots of the provider COCI and site COCI: (**a**) raw data and (**b**) data obtained after log transformation.

COC was calculated using the Continuity of Care Index (COCI). The mean provider COCI in patients with ESRD during the 6–12 months before death was 0.27, and the distribution was right-skewed (Fig. 1a, left). Only 2% of the patients experienced perfect provider continuity (provider COCI = 1) in our sample (Fig. 1a, left). The distribution of the provider COCI was normalized through log transformation (Fig. 1b, left). The site COCI, which had a mean value of 0.59, was considerably higher than the provider COCI. The distribution of the site COCI differed from the distribution of the provider COCI, with the overall distribution of the site COCI being relatively even before (Fig. 1a, right) and after log transformation (Fig. 1b, right).

### COC and health expenditures

Our analyses revealed that better COC would result in lower cumulative expenditures in the 3 and 6 months before the deaths of patients with ESRD (Table 2). Each 1% increase in the provider COCI was associated with an 8% [95% confidence interval (CI) 7–9%] and a 6% (95% CI 4%–7%) reduction in the total health costs in the 6 and 3 months before death, respectively. We did not detect statistically significant effects of site COC on cumulative expenditures (Table 2).

**Table 2:** Predictors of health expenditure within 6 and 3 months before death (log-transformed COCI).

	6 months before death		3 months before death	
	Exponentiated coefficients	95% CI	Exponentiated coefficients	95% CI
Provider COCI	0.92***	(0.91–0.93)	0.94***	(0.93–0.96)
Site COCI	0.99	(0.97–1.01)	0.99	(0.97–1.01)
**Patient characteristics**				
Sex (control: male)	0.99	(0.98–1.01)	0.97***	(0.95–0.99)
Comorbidity (CCI)	1.05***	(1.05–1.05)	1.04***	(1.04–1.05)
Age at death	1.00***	(0.99–1.00)	0.99***	(0.99–1.00)
Insurable monthly earnings	1.00	(1.00–1.00)	1.00	(1.00–1.00)
Peritoneal dialysis (control: hemodialysis)	1.04	(0.99–1.09)	1.07*	(1.01–1.14)
**Physician characteristics: visited the most for 6 months to one year before death**				
Physician sex (control: male)	1.00	(0.97–1.02)	0.99	(0.95–1.02)
Physician age	1.00	(1.00–1.00)	1.00	(1.00–1.00)
Physician Specialty: Internal medicine (control: nephrologist)	0.97*	(0.95–0.99)	0.97*	(0.94–1.00)
Physician specialty: family medicine (control: nephrologist)	0.95*	(0.91–0.99)	0.94*	(0.89–1.00)
Physician specialty: surgeon (control: nephrologist)	1.01	(0.98–1.04)	1.01	(0.97–1.05)
Physician specialty: others (control: nephrologist)	0.97*	(0.95–1.00)	0.97	(0.94–1.00)
**Hospital characteristics: visited the most for 6 months to one year before death**				
Private hospitals (control: public)	0.99	(0.97–1.01)	1.00	(0.97–1.02)
Regional hospitals (control: medical centers)	0.98	(0.96–1.01)	0.99	(0.96–1.02)
Local hospitals (control: medical centers)	0.95***	(0.92–0.97)	0.93***	(0.90–0.96)
Local clinics (control: medical centers)	0.93***	(0.91–0.96)	0.94***	(0.91–0.97)
Homecare (control: Medical Centers)	0.75*	(0.57–0.98)	0.65*	(0.43–1.00)
Observations	29,095		29,095	

Data are presented as exponentiated coefficients (95% CI). All the analyses were controlled for year of death and region.

*COCI* Continuity of Care Index, *CI* confidence interval, *CCI* Charlson Comorbidity Index.

***p < 0.001, **p < 0.01, *p < 0.05.

**Figure 2:**
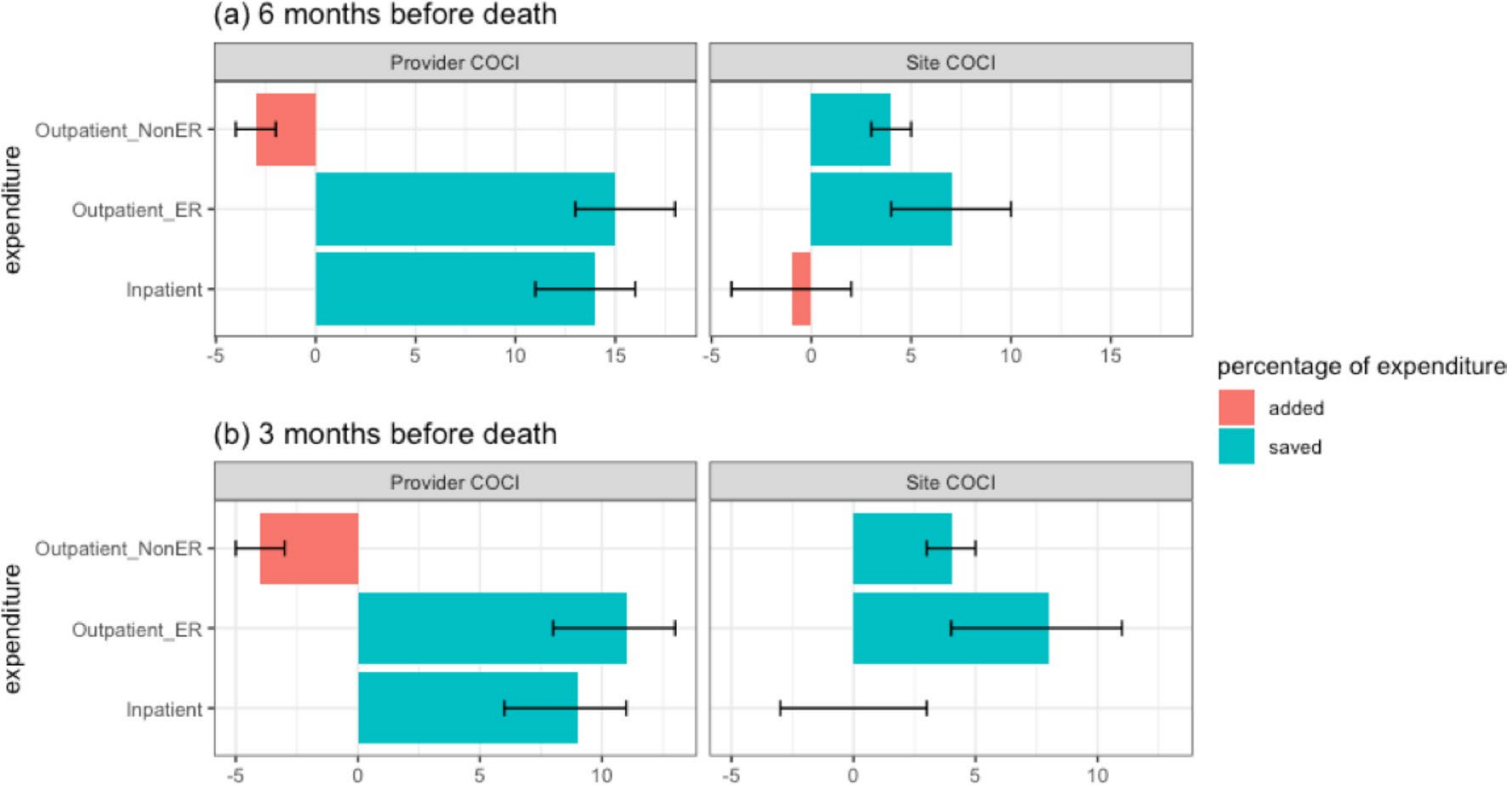
Percentage of expenditure added and saved after a 1% change in the provider COCI and site COCI: (**a**) 6 months before death and (**b**) 3 months before death (data divided by categories and displayed with 95% CI).

A thorough comparison across the different categories of expenditures reduced by a 1% increase in the COCI is displayed in Fig. 2. This comparison revealed that the reduction in expenditures resulted principally from savings on inpatient services and emergency room (ER) visits. Expenditures from regular outpatient visits increased as the provider COC increased (Fig. 2). A deep examination of the effect of COC on different categories of inpatient expenditures suggested that the savings were the result of lower procedure and drug fees (Table 3).

**Table 3:** Odds ratio from fractional probit model predicting different categories of inpatient expenditures, with the total inpatient expenditure as the denominator.

	Continuity of Care Index-provider		Continuity of Care Index-site	
	OR	95% CI	OR	95% CI
**6 months before death**				
Diagnosis	1.02***	(1.01–1.04)	1.01*	(1.00–1.02)
Room	1.03*	(1.00–1.05)	1.02*	(1.00–1.04)
Check up	1.09***	(1.06–1.11)	0.97**	(0.96–0.99)
Radiotherapy	1.05*	(1.00–1.11)	0.93***	(0.90–0.97)
Procedure	0.96**	(0.93–0.99)	1.07***	(1.05–1.09)
Surgery	1.00	(0.94–1.07)	0.88***	(0.84–0.93)
Drug	0.98	(0.95–1.01)	0.99	(0.97–1.01)
**3 months before deaths**				
Diagnosis	1.03***	(1.01–1.05)	1.01	(1.00–1.02)
Room	1.03*	(1.00–1.06)	1.02	(1.00–1.04)
Check up	1.06***	(1.04–1.10)	0.97**	(0.95–0.99)
Radiotherapy	1.06*	(1.01–1.12)	0.95**	(0.91–0.99)
Procedure	0.97	(0.94–1.00)	1.06***	(1.04–1.08)
Surgery	0.98	(0.91–1.06)	0.90***	(0.85–0.95)
Drug	0.97*	(0.94–1.00)	0.99	(0.97–1.01)

*OR* odds ratio, *CI* confidence interval.

### Association between COC and hospice palliative care, utilization of acute care, and invasive treatments

Table 4 indicates that after controlling for the site COCI, patients with perfect provider COCI (provider COCI = 1) had an odds ratio (OR) of 0.78 in the utilization of the intensive care unit (ICU) compared with patients with the worst provider COCI (provider COCI = 0). Moreover, after controlling for the provider COCI, patients with perfect site COCI had an OR of 0.72 in the utilization of the ER compared with patients with the worst site COCI. Furthermore, high provider COC decreased the utilization of mechanical ventilation (OR 0.72, 95% CI 0.62–0.84), continuous renal replacement therapy (CRRT) (OR 0.72, 95% CI 0.52–0.99), nasogastric (NG) intubation (OR 0.74, 95% CI 0.63–0.86), and surgical intervention (OR 0.73, 95% CI 0.62–0.86) in the last 30 days before death. Higher site COC resulted in lower odds of surgical interventions (OR 0.83, 95% CI 0.74–0.94) and ER visits (OR 0.74, 95% CI 0.67–0.83) but higher odds of NG intubation in the last 30 days before death (OR 1.13, 95% CI 1.02–1.27). Only 529 patients (1.82%) in our sample received hospice palliative care during the last year before death, and the rate of hospice palliative care was not statistically correlated with provider or site COC.

**Table 4:** ORs from logistic regressions predicting acute care, hospice palliative care, and invasive interventions utilizations.

	Continuity of Care Index- provider		Continuity of Care Index- site	
	OR	95% CI	OR	95%CI
**Hospice palliative care**				
Hospice palliative care	0.80	(0.42–1.55)	1.11	(0.75–1.66)
**Acute care**				
ER visit	0.88	(0.76–1.02)	0.74***	(0.67–0.83)
ICU	0.78***	(0.67–0.90)	0.95	(0.86–1.06)
**Invasive interventions**				
Ventilation	0.72***	(0.62–0.84)	0.96	(0.87–1.07)
CPR	0.92	(0.76–1.11)	1.01	(0.88–1.16)
Endotracheal intubation	0.90	(0.77–1.06)	1.02	(0.90–1.14)
Continuous renal replacement therapy	0.61***	(0.44- 0.84)	0.84	(0.68–1.05)
NG tube	0.74***	(0.63–0.86)	1.13*	(1.02–1.27)
Surgery	0.73***	(0.62–0.86)	0.83***	(0.74–0.94)

*OR* odds ratio, *CI* confidence interval.

## Discussion

The findings of this study affirmed the importance of COC in enhancing the quality of end-of-life care. Our findings are in agreement with those of previous studies that have reported that even minor changes in the COC noticeably affect expenditures for patients with diabetes, chronic heart failure, and chronic obstructive pulmonary disease patients^17^. We also observed that changes in provider COC had a greater effect on the end-of-life care expenditure than changes in site COC did. Furthermore, high COC was associated with a reduction in the utilization of high-intensity healthcare services and invasive treatments, such as ICU admissions, ER visits, ventilators, CRRT, and surgical interventions, in the last 30 days.

Several mechanisms can explain the observed effects of COC on end-of-life care expenditures. First, high provider COC indicates an established patient–provider relationship, which allows physicians to prevent unnecessary admissions. Therefore, patients are treated predominantly in outpatient settings^21^. Second, high provider COC leads to accrued knowledge between patients and physicians, which may prevent wasteful or duplicative drug prescriptions and procedures^16^. This hypothesis was confirmed in the fraction analysis of various categories of inpatient expenditures.

While high provider COC displayed significant and substantial effects on reducing health expenditures, site COC had less influence than provider COC on the aforementioned parameters. We hypothesize that higher site COC may indicate more coordinated care from various specialists^22^, which reduces ER and non-ER outpatient expenditures. High site COCI is not statistically associated with lower total and inpatient expenditures, which indicates that loss of information may occur despite health records being shared between physicians within the same facility. This phenomenon may result from uncoordinated care among providers. We conclude that location continuity alone is insufficient to generate the benefits of clinical continuity offered by a primary physician^23^. Future studies should prioritize the development of coordinated informatics to facilitate high-quality end-of-life care.

In contrast to our hypothesis, neither higher provider COC nor higher site COC statistically increased the enrollment into hospice palliative care. These results may have been caused by the extremely low hospice palliative care enrollment rate in Taiwan and the time required for the palliative care policy to be fully executed. A study investigated the cultural differences between Taiwan and Western countries on attitudes and perceptions toward end-of-life care^24^, and displayed that countries where individualism is valued more than collectivism prioritize the autonomy of patients for end-of-life care. Although patients in Taiwan share the same attitude of rejecting futile treatments to prolong life as patients in countries such as New Zealand^24^, obstacles remain in implementing patients’ wishes. Patients and their families refrain from discussing end-of-life care because this topic is perceived as a jinx. Numerous families dictate whether physicians can disclose the prognosis to patients and make medical decisions on behalf of dying family members^25^; Furthermore, social norms in Taiwan perceive people as unfilial if they reject life-sustaining treatments for elder members of the family^26^.

Consequently, families in Taiwan are more likely to make conservative medical decisions (e.g., continuing mechanical ventilators), and physicians are reluctant to take a proactive role in end-of-life decisions or discontinue invasive life-sustaining treatments. Our study revealed that 46.14% of patients with ESRD had utilized mechanical ventilators during the last 30 days of life, which aligns with research in Taiwan that reported that 45.3% of elderly patients with chronic kidney disease used mechanical ventilators before death^27^. These statistics are considerably larger than the corresponding ones for elderly patients with ESRD in the United States (22.2%)^12^. The unpredictable disease trajectory of ESRD also makes it difficult for physicians to determine the timing for hospice palliative care because the most common cause of death in patients with ESRD is cardiac arrest^28,29^. Finally, our study period (2005–2013) might have been unsuitable for observing the effect of providing hospice palliative care for patients with ESRD because this type of care was introduced in Taiwan in 2009.

Studies have demonstrated that site COC reduces ER visits for patients with multiple comorbidities and ICU utilization for lung cancer patients^19,22^. This finding is in agreement with our findings for patients with ESRD. The aforementioned phenomena may result from the higher access to medical information on patient conditions for patients with higher site COC^30^. Furthermore, high provider COC decreased the utilization of ventilators, NG intubation, and CRRT. The reduction in CRRT strongly indicates that continuous caring relationships between physicians and patients with ESRD should be promoted.

Research on end-of-life quality of care has generally been confined to cancer patients. Our population-based study is the first to examine COC among elderly patients with ESRD and its effects on end-of-life quality of care. We utilized a broad range of quality of life indicators, including expenditures, hospice palliative care enrollment, utilization of acute care, and intensive treatments.

However, actual disease severity was not recorded in our dataset, which could bias our results of ER visits and ICU admissions. Disease severity may also affect COC, but the direction of the effect requires further research. To address this problem, we controlled for comorbidity index, and the prognosis among our study population was relatively homogenous because they all died within 1 year. We were also unable to obtain data on the family network and other social determinants of health from the claims datasets, which could affect the outcome measures. Finally, do-not-resuscitate (DNR) could be an important mediator for the reduction of healthcare expenditure in the present study. Unfortunately, information of DNR orders is not available in our data. It would be valuable for future studies to use electronic medical records to examine the impact of continuity of care on DNR for policy guidance.

The external validity of our research may be restricted to healthcare systems similar to the one in Taiwan, where a referral system is not fully established. Furthermore, we evaluated COC and quality of care. However, end-of-life care should focus on the comfort of patients and symptom control. Therefore future end-of-life research should employ both claims data and subjective patient experiences to obtain a deeper understanding of the problems with end-of-life quality of care.

In addition to confirming the importance of COC, we determined that provider COC was more beneficial than site COC for patients with ESRD in end-of-life care. Therefore, we suggest promoting a primary care physician model in addition to the current policy focus on coordinated care. To improve the quality of care, primary care physicians can initiate discussions on end-of-life care and coordinate with other departments. Our findings and recent policy changes present several research opportunities to investigate quality of care, which may improve the efficiency of the healthcare system and the quality of care for patients with ESRD.

## Materials and methods

### Setting and participants

The research data were obtained from the National Health Insurance Research Database (NHIRD) in Taiwan^31^. The NHIRD is a claims database that covers more than 99% of the residents in Taiwan. Patients were assigned a unique identifier to link datasets in the NHIRD, such as the data on basic demographic information and medical claims. Outpatient and inpatient records were mainly used, including information on the primary and secondary diagnosis, date of visit, length of stay, procedures performed, expenditure filed for reimbursement (in total and by categories), and drugs prescribed. Patients with catastrophic illness certificates that exempt them from all copayments of visits related to their specific conditions are marked in the NHIRD. Certain illnesses, such as cancer, organ transplant, and ESRD, entitle patients to apply for the catastrophic illness certificate^32^.

### Study population

A retrospective cohort design was used to assess the effect of COC on end-of-life quality of care among patients with ESRD. The NHIRD’s catastrophic illness file was used to identify the sample population, which comprised elderly patients (≥ 65 years old) who were diagnosed as having ESRD for at least 1 year before death from 2005 to 2013 in Taiwan. Kidney transplant recipients and patients with fewer than three outpatient visits within the 6–12 months before death were excluded.

This study focused on the elderly population because of the disease burden caused by an aging society on Taiwan’s healthcare system and to ensure similar disease patterns across the study population. Young patients with ESRD may display different disease etiologies. Among young patients, congenital anomalies are the most common causes of ESRD. By contrast, in most patients aged over 65 years old, diabetes and hypertension are the main causes of ESRD^33^. Furthermore, the study population was confined to patients with catastrophic illness certificates in the last year before death because the issuance of these certificates may affect COC. Catastrophic illness certificates guarantee exemption from copayments, which may influence health-seeking behaviors by intensifying “doctor shopping.”

Patients who had undergone kidney transplant surgery were excluded because their death rates were considerably lower than those of patients receiving dialysis, which may affect the provider’s treatment plan^33^. Finally, patients with fewer than three outpatient visits in the 6–12 months before death were excluded on the basis of previous studies on COC^15,34,35^ that have reported that COC has little effect on patients who seldom utilize outpatient care.

### Primary predictors

The key independent variable was the COC from 6–12 months before death. Common indices used in research on claims datasets are as follows: the usual provider of care index, which measures the density of visits to a physician; the COCI, which measures the dispersion of visits; and the sequential continuity index, which recognizes consistent providers when seen sequentially^20,36^. The NHIA does not apply a gatekeeping policy in Taiwan. Therefore, patients are free to visit the physician of their choice. This freedom results in large variations in and an overall high number of physician visits. The COCI is less sensitive to the number of physician visits than the other two indices are^37^; thus, the COCI is the most suitable index for this research^15,20^. The COCI of each person was calculated using the following equation^22^:

$${\text{Provider}}\;{\text{ COCI}} = \frac{{\left( {\sum _{{{\text{i = 1}}}}^{{\text{p}}} n_{i}^{2} } \right) - T}}{{T(T - 1)}}$$

$${\text{Site}}\;{\text{COCI}} = \frac{{\left( {\mathop \sum \nolimits_{{j = 1}}^{q} n_{j}^{2} } \right) - T}}{{T\left( {T - 1} \right)}}$$

where $T$ is the total number of outpatient visits, ${n}_{i}$ is the total number of visits to physician $i$, $p$ is the total number of physicians, $q$ is the number of sites, and ${n}_{j}$ is the number of visits to the same site $j$. The COCI ranges from 0 to 1, with 0 indicating no continuity and 1 indicating perfect continuity. For each outpatient visit, only one provider claim was included. If a patient visited two or more physicians on the same day, the claims were listed as separate records. The same rule was applied to referred visits. Peritoneal dialysis, which is performed at home by patients, was not documented in the claims data and thus was not included in outpatient visits. Visits to dentists or Chinese medicine practitioners were excluded to increase the comparability of the COCI^20^.

The site COCI was calculated in addition to the provider COCI because medical records are accessible across departments in the same facility. Therefore, patients with high site COCI may share similar benefits to those with high provider COCI. Thus, identifying the separate effects of the provider COCI and site COCI on the quality of care is crucial.

### Outcome

**Figure 3:**
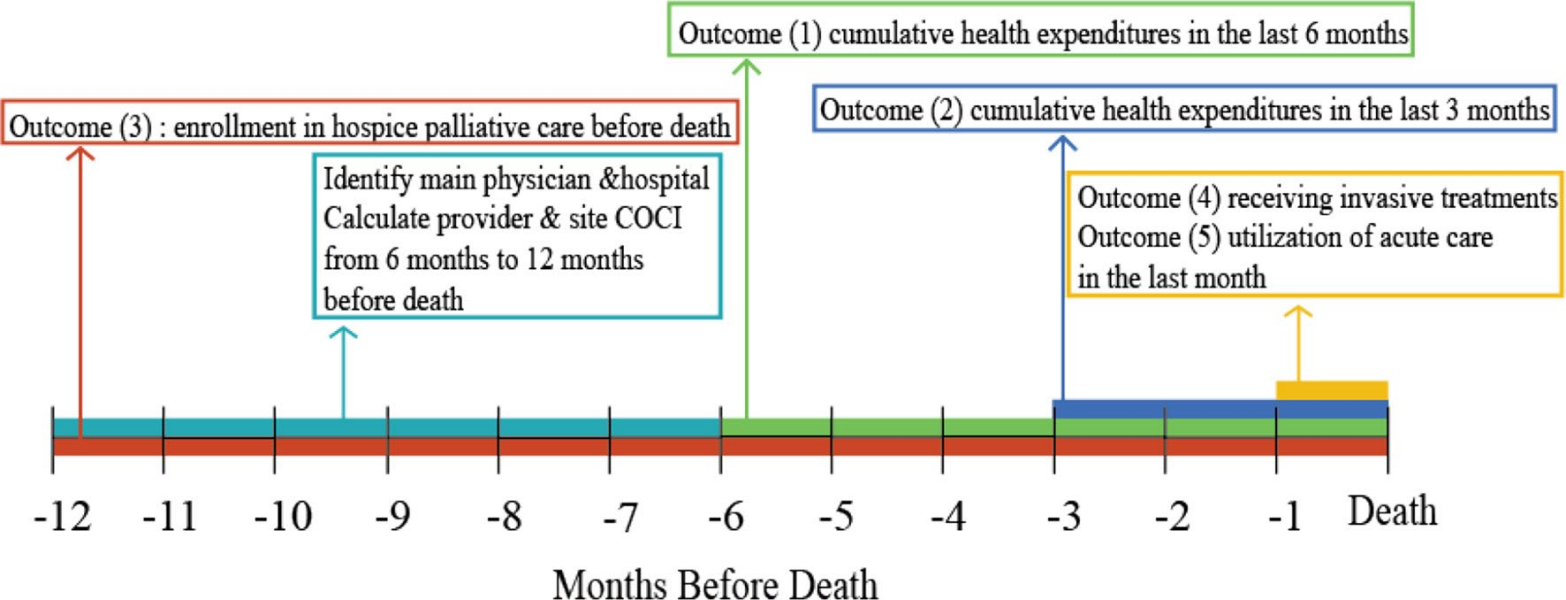
Outcome variables on a timeline before death.

The outcome variables are displayed in Fig. 3.

Expenditures were used as the primary indicators because high expenditures during the end of life are associated with inferior quality of care and increased aggressiveness of care^38,39^. Nominal expenditures in New Taiwan Dollars were used because the reimbursement is fixed based on a fee schedule that has not been updated for inflation.

Enrollment in hospice palliative care is strongly recommended for patients with ESRD because of the foreseen unfavorable outcomes^40^. Enrollment in hospice palliative care serves as consent for providers to change treatment schemes from disease-centered care to palliative care, which focuses on pain and symptom control. Therefore, the quality of end-of-life hospice palliative care is considered higher than that of disease-centered care.

Utilization of acute care is defined as admissions to the ICU or visits to the ER in the last month before death. Both measures have been routinely utilized in past research to measure the intensity of end-of-life care^41^. A literature review^4,27,41^ revealed that the following treatments are considered invasive end-of-life care: resuscitation, surgical interventions, mechanical ventilation, NG intubation, and endotracheal intubation. Binary variables were generated for each treatment to determine whether the patients received a treatment in the last 30 days before death.

The utilization of CRRT was used as an indicator of overtreatment during end-of-life care. CRRT is a common practice in critical nephrology for patients with unstable hemodynamics, such as shock or multiple organ failure. Although dialysis discontinuation is recognized as an appropriate treatment option in end-of-life care for patients with ESRD^40^, this option is rarely adopted because of treatment norms, the lack of financial barriers, and patients’ unfamiliarity with it^42^. Therefore, dialysis continuation at the end of life was not used as a measure of poor care quality. However, receiving CRRT, which is a life-sustaining procedure that is usually performed on critically ill patients with multiple organ failure^43^, was used for accurately indicating poor quality of end-of-life care among patients with ESRD.

### Independent variables

Patient sex, age, beneficiary earnings, and comorbidity in terms of the Charlson Comorbidity Index were controlled at the individual level^44^. If an elderly patient was a dependent under a policyholder, then the primary policyholder’s earnings were used^20^. Patients were considered to have a comorbid condition if they had two outpatient visits or one inpatient record with an ICD-9 code for that condition in their final year before death. Other independent variables controlled were the year of death and the use of peritoneal dialysis.

The main physician was identified by calculating the highest number of visits each patient made to a physician during the 6–12 months before death (Fig. 3). The main physician’s age, sex, and specialty were included in the regression model as covariates. The main hospital was identified using the same process (most visited healthcare facility 6–12 months before death). The teaching status of a health facility was included in the analysis when outcome variables concerned inpatient care expenditure, hospice palliative care, acute care, and invasive interventions. Regional differences were controlled using the six-region classification of the National Institutes of Health.

### Analytic approach

Generalized linear models were employed to analyze the determinants of health expenditure by using the log-transformed site and provider COCIs as key independent variables. Because health expenditures were skewed and always positive, gamma distributions with a log link were used in the models. Gamma distributions assume that variance is proportional to the square of the mean. The exponentiated coefficients were determined because they could be directly interpreted as the factor by which the mean costs in the reference group were multiplied.

Multivariate logistic regression models were used with raw site and provider COCIs as key independent variables for all the other outcome variables, including hospice palliative care enrollment, utilization of acute care, and intensive treatments. All the models used robust standard errors, and the OR and 95% CI of the predictors were determined. A *p* value of less than 0.05 was considered statistically significant. All the analyses were performed using STATA version 15 (StataCorp LP, College Station, Texas, USA).
